# Edwin Vincent Foss

**Published:** 1935

**Authors:** 


### EDWIN VINCENT FOSS, M.B., CH.B. (BRISTOL), M.R.C.S., L.R.C.P.

EDWIN VINCENT FOSS was born in 1872 in Bristol. A studious youth, he won an Open Scholarship for Queen Elizabeth's Hospital in 1883. From here he won a scholarship when fifteen years of age to the Grammar School. He started his medical training at the Bristol Medical School at the age of nineteen years, and his clinical teaching was obtained at the General Hospital under Dr. Michell Clarke, Mr. Morton and Mr. Firth. He showed great keenness and proficiency, and was awarded the Committees' Gold Medal and the Lady Haberfield, Clarke, Sanders, and Martyn Scholarships.

He qualified M.R.C.S., L.R.C.P., in October, 1898, and started at once in General Practice with Dr. James Young at St. George. Dr. Foss soon won the respect and devotion of the people of St. George, and appeared not to miss the usual Hospital appointments after qualification. His careful and painstaking efficiency stood him in good stead, and he gradually built up a very large practice in East Bristol. He was appointed District Medical Officer to the old Bristol Guardians in 1906 and Public Vaccinator to District 5, and District Medical Officer to the Post Office and Education Department in 1907. He managed to keep his book work up to date, and in 1912 he obtained the Bristol M.B., Ch.B., and was appointed

Medical Officer to the East Division of the Bristol Tramways and Carriage Company in 1916. He was also Medical Officer for the Juvenile Foresters and Medical Referee for the Pearl and other Assurance Companies. These appointments he held until he retired two years before his death.

He married in 1906, and had seven children. As a young man he was an enthusiastic Freemason, being a Past Master of the Yatton Lodge and a Founder of the Robert Thorne Lodge. Later his family and his profession absorbed the whole of his time. In his odd moments he loved his bees and flowers. During the influenza epidemic of 1932 he fell himself a victim to the infection, but carried on working all hours, and gradually his health began to fail. His last years were full of suffering, and he died on 11th November, 1935, after a long illness.

